# Metabolically healthy obesity and risk of incident type 2 diabetes: a meta-analysis of prospective cohort studies

**DOI:** 10.1111/obr.12157

**Published:** 2014-03-24

**Authors:** J A Bell, M Kivimaki, M Hamer

**Affiliations:** Department of Epidemiology & Public Health, University College LondonLondon, UK

**Keywords:** Metabolic health, obesity, type 2 diabetes

## Abstract

The risk of type 2 diabetes among obese adults who are metabolically healthy has not been established. We systematically searched Medline (1946–August 2013) and Embase (1947–August 2013) for prospective studies of type 2 diabetes incidence (defined by blood glucose levels or self-report) among metabolically healthy obese adults (defined by body mass index [BMI] and normal cardiometabolic clustering, insulin profile or risk score) aged ≥18 years at baseline. We supplemented the analysis with an original effect estimate from the English Longitudinal Study of Ageing (ELSA), with metabolically healthy obesity defined as BMI ≥ 30 kg m^−2^ and <2 of hypertension, impaired glycaemic control, systemic inflammation, adverse high-density lipoprotein cholesterol and adverse triglycerides. Estimates from seven published studies and ELSA were pooled using random effects meta-analyses (1,770 healthy obese participants; 98 type 2 diabetes cases). The pooled adjusted relative risk (RR) for incident type 2 diabetes was 4.03 (95% confidence interval = 2.66–6.09) in healthy obese adults and 8.93 (6.86–11.62) in unhealthy obese compared with healthy normal-weight adults. Although there was between-study heterogeneity in the size of effects (*I*^2^ = 49.8%; *P* = 0.03), RR for healthy obesity exceeded one in every study, indicating a consistently increased risk across study populations. Metabolically healthy obese adults show a substantially increased risk of developing type 2 diabetes compared with metabolically healthy normal-weight adults. Prospective evidence does not indicate that healthy obesity is a harmless condition.

## Introduction

The global burden of type 2 diabetes is building dramatically, with upwards of 370 million people estimated to have diabetes; half of whom may be unaware of their condition (1). Obesity is a well-established risk factor for type 2 diabetes (2). Although histological characteristics of adiposity play a direct role (3), much of the increased risk for diabetes among the obese is thought to stem from the underlying cardiometabolic abnormalities associated with excess fat, such as islet beta-cell dysfunction, insulin resistance, hyperglycaemia (3) and high chronic systemic inflammation (4,5). Other contributing factors may include higher levels of visceral fat (6), an energy-dense/nutrient-poor diet including excessive sugar intake (7–9), and physical inactivity (10,11) along with genetic, ethnic and socioeconomic susceptibilities (12,13).

However, not all obese individuals seem to carry such risk. Nearly one-third of obese adults in the general population are considered metabolically healthy (14,15) and display favourable levels of biological factors relevant to type 2 diabetes development. These include normal insulin sensitivity, normoglycaemia, low inflammation (16,17) and higher cardiorespiratory fitness (18). It remains unclear if obese adults who are metabolically healthy also face an increased risk for type 2 diabetes over time. Given the increasing prevalence of obesity worldwide (19), a better understanding of the health consequences facing its distinct phenotypes would benefit both public health and clinical practice, as well as support more efficient strategies for type 2 diabetes prevention and management.

To facilitate this, the objective of this study was to synthesize existing prospective evidence on the risk of incident type 2 diabetes for metabolically healthy obese adults in a meta-analysis, supplemented by original individual-level data obtained from a nationally representative sample of older adults in England.

## Methods

### Meta-analysis of published cohort studies

#### Data sources and searches

A meta-analysis was conducted according to the Meta-analysis of Observational Studies in Epidemiology (MOOSE) criteria (20). An OvidSP-led systematic search of Medline (date range: from 1946 to August 2013) and Embase (date range: from 1947 to August 2013) was performed in August 2013 by JAB. Truncated search terms included ‘obese’, ‘body mass index’, ‘metabolic’, ‘diabetes’, ‘type 2’, ‘risk’ and ‘incidence’. No language restrictions were applied.

#### Study selection

JAB and MH independently screened search results and agreed on studies to be included. Abstracts were scanned, and references within relevant papers were hand-searched for additional works. Studies were eligible for inclusion in the meta-analysis if they met the full pre-specified criteria for exposure (metabolically healthy obesity defined by body mass index (BMI) and normal cardiometabolic clustering, insulin profile or risk score), outcome (type 2 diabetes incidence defined by blood glucose levels or self-report), population (adults aged ≥18 years at baseline) and study design (original prospective estimation).

#### Data extraction and quality assessment

As estimates may be presented at more than one stage of statistical adjustment, we elected to use fully adjusted estimates for analyses, as these were more likely to be the closest approximations of true study effects and offer more comparability between studies. Study quality was assessed according to the rigor of the study's exposure, outcome and model adjustment strategy. Regarding the exposure, 2 points were assigned if the study considered metabolic clustering and 1 point if the study considered insulin profile alone. For the outcome, 2 points were assigned if the diagnosis was based upon an objective clinical measurement (i.e. fasting plasma glucose), and 1 point if the diagnosis was based only upon self-report. Based upon the suggested importance in the literature, studies were assigned 1 point if they considered each of the following relevant covariates: family history of diabetes, ethnicity, alcohol consumption, smoking status, physical activity/cardiorespiratory fitness, dietary sugar intake and socioeconomic status. Studies were therefore scored out of 11 points, with higher scores reflecting better study quality.

### The English Longitudinal Study of Ageing

We supplemented studies identified through the literature search with individual-level data from the English Longitudinal Study of Ageing (ELSA). ELSA is an ongoing cohort of a nationally representative sample of men and women born on or before 29 February 1952 living in private households in England (21). The sample was drawn using multistage stratified probability sampling with postcode sectors selected at the first stage and household addresses selected at the second stage. Data from wave 2 (2004–05) when participants were 52 years or older were used as the baseline. Participants gave full informed written consent to participate in the study and ethical approval was obtained from the London Multi-Centre Research Ethics Committee.

Nurses collected anthropometric data (weight and height), measured blood pressure (BP) and took blood samples, as previously described (22). Blood samples were analysed for C-reactive protein (CRP), high-density lipoprotein (HDL)-cholesterol, triglycerides and glycated haemoglobin (HbA1c). BMI was calculated using the standard formula (weight [kilograms]/height [meters] squared). Normal-weight (BMI < 25 kg m^−2^), overweight (BMI 25 < 30 kg m^−2^) and obese (BMI ≥ 30 kg m^−2^) were defined using conventional criteria. ‘Metabolically healthy’ status was defined as <2 of the following metabolic risk factors: hypertension (clinic BP > 130/85 mmHg, or hypertension diagnosis, or use of anti-hypertensive medication), impaired glycaemic control (HbA1c > 6.0%), systemic inflammation (CRP ≥ 3 mg L^−1^), adverse HDL-cholesterol (<1.03 mmol L^−1^ in men and <1.30 mmol L^−1^ in women) and adverse triglycerides (≥1.7 mmol L^−1^), based upon comprehensive criteria (14) that have been previously employed in ELSA (22). Participants were categorized into six groups: ‘metabolically healthy normal-weight’; ‘metabolically unhealthy normal-weight’; ‘metabolically healthy overweight’; ‘metabolically unhealthy overweight’; ‘metabolically healthy obese’; and ‘metabolically unhealthy obese’.

Type 2 diabetes was recorded from self-reported physician diagnosis, which has been previously validated in ELSA (23). Incident cases of diabetes were recorded over waves 3 (2006/2007), 4 (2008/2009) and 5 (2010/2011), thus follow-up ranged from 2 to 6 years (mean = 5.9 years). Participants with type 2 diabetes at baseline were excluded from analyses.

Demographic and health-related questions included cigarette smoking (current, previous or non-smoker), the frequency of participation in vigorous, moderate and light intensity physical activities (more than once per week, once per week, one to three times per month, hardly ever), and frequency of alcohol intake (daily, 5–6/week, 3–4/week, 1–2/week, 1–2/month, once every couple of months, 1–2/year, never). Depressive symptoms were assessed using the 8-item Centre of Epidemiological Studies Depression scale. Wealth served as a comprehensive measure of socioeconomic status, calculated as net of debt and included the total value of the participant's home (excluding mortgage); financial assets such as savings, business assets; and physical wealth such as artwork or jewellery.

#### Data synthesis and analysis

In analysing data from ELSA, we used Cox proportional hazard models to compute hazard ratios (HRs) with accompanying 95% confidence intervals (CIs) for the association of metabolic health/obesity categories with diabetes. The proportional hazard assumption was examined by comparing the cumulative hazard plots grouped on the various exposure variables, although no appreciable violations were noted. Years of follow-up were the time scale, and for participants with no record of an event, the data were censored at wave 5 (maximum 6 years follow-up). Models were adjusted for age, sex, and behavioural and socio-demographic covariates, including smoking, alcohol, physical activity, depressive symptoms and wealth quintile. This modelling strategy was planned *a priori* based upon existing evidence linking these covariates with obesity and diabetes (24–27). Analyses were conducted using SPSS, version 21 (SPSS Inc., Chicago, IL, USA).

We used meta-analysis to synthesize data from published studies identified through the literature search and ELSA. Natural variation in study effects was expected due to differences in such factors as obesity phenotype definition, sampling procedure, statistical adjustment strategy and sample demographics. A random effects model was therefore employed to estimate the mean of the distribution of effects, with the *I*^2^ statistic used to describe the percentage of between-study heterogeneity (28). Odds ratios (ORs), HRs and relative risk (RR) ratios were pooled and log-transformed for analyses. Random effects meta-regression was planned *a priori* to examine the extent to which age, ethnicity, duration of follow-up and study quality including phenotype criteria explain any observed between-study heterogeneity in effects. The meta-analysis was performed using Stata 12 (StataCorp, College Station, TX, USA).

## Results

### Literature search of published studies

**Figure 1:**
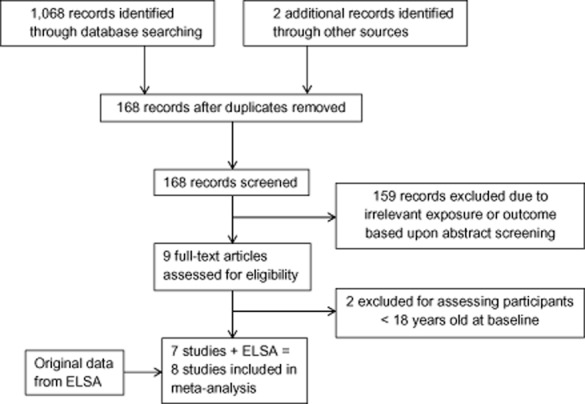
Outline of the systematic study selection process for the meta-analysis.

As shown in Fig. 1, the initial search of Medline and Embase retrieved 1,068 results. Two additional studies were identified through other sources (29,30). After removing duplications, 168 studies remained, 159 of which were excluded due to irrelevant exposure or outcome based upon abstract screening. Nine studies were identified after screening as potentially relevant, and full-text articles were assessed for eligibility. Of these, two studies were excluded for assessing participants less than 18 years of age at baseline (31,32). Seven published studies therefore met our full criteria for inclusion (29,30,33–37). Hand-searching through reference lists within those seven included studies identified six additional potentially relevant studies, but none of these met the full inclusion criteria. For instance, one study assessed cross-sectional type 2 diabetes prevalence but not prospective incidence (38).

### The English Longitudinal Study of Ageing

The sample comprised 3,066 individuals (aged 64.6 ± 8.5 years, 43.3% men). Compared with the metabolically unhealthy obese and non-obese, metabolically healthy obese participants were on average younger, less likely to be smokers and had intermediate levels of risk factors (see Supporting Information Appendix S1). The metabolically healthy obese had lower BMI than their unhealthy obese counterparts.

Over 6 years of follow-up, there were 138 incident diabetes cases. Relative to healthy normal-weight participants, the highest risk of incident diabetes was observed in the unhealthy obese, followed by the unhealthy overweight and unhealthy normal-weight (Supporting Information Appendix S2). The metabolically healthy obese were also at elevated risk (HR = 8.6; 95% CI = 2.4–30.4) after adjustment for behavioural and socio-demographic covariates. The unhealthy obese had a substantially higher risk of diabetes than the metabolically healthy obese participants despite adjustment for all covariates, including wealth and health behaviours (HR = 23.5; 95% CI = 7.3–75.6). The metabolically unhealthy overweight (HR = 16.7; 95% CI = 5.2–54.2) and the unhealthy normal-weight (HR = 9.9; 95% CI = 2.9–36.7) were also at higher risk of incident diabetes than the metabolically healthy normal-weight participants. The pattern of results across groups remained the same when metabolically healthy phenotypes were defined as having zero metabolic abnormalities (Supporting Information Appendix S3).

### Meta-analysis of published cohort studies and English Longitudinal Study of Ageing

Including ELSA, a total of eight studies contributed to the meta-analysis. Two studies reported the effect estimates for type 2 diabetes separately by sex (30,34), and were presented accordingly. The studies included in the meta-analysis represented a geographically diverse set of populations (Table 1); however, ethnic composition was not specifically reported in any study. Age at baseline ranged from 18 years in three studies (29,34,37) to 99 years in ELSA. All studies defined metabolic health based upon metabolic clustering, with the least comprehensive measure considering only insulin resistance, triglycerides and fasting glucose (37). All studies used an objective fasting blood or plasma glucose measure to diagnose incident type 2 diabetes except for ELSA, which used self-reported physician diagnosis. Average length of follow-up ranged from 5 years in Kim *et al*. (35) to 20 years in Arnlov *et al*. (33).

**Table 1:** Characteristics of studies included in the meta-analysis

Study	Baseline sample	Baseline healthy obese *n* (% of obese)	Metabolically healthy obese criteria	Type 2 diabetes criteria	Follow-up length Diabetes cases for healthy obese	Estimate (95% CI)	Covariates	Quality score (/11)*
Meigs *et al*. 2006 (36)	*n* = 2,902 Mean 52 years (MHO group) 51.3% male (MHO group) Free of type 2 diabetes or CVD United States Community-based	236 (37%)	Criteria 1: ≤2 ATP3 metabolic syndrome criteria: fasting plasma glucose 5.6–6.9 mmol L^−1^, waist circumference > 102 cm in men or >88 cm in women, fasting triglycerides ≥ 1.7 mmol L^−1^, HDL-cholesterol < 1.0 mmol L^−1^ in men or <1.3 mmol L^−1^ in women, blood pressure ≥ 130/85 mmHg or current treatment for hypertension Criteria 2: HOMA-IR ([Fasting glucose × Fasting insulin]/22.5) <75th percentile BMI ≥ 30 kg m^−2^	Fasting plasma glucose ≥ 7.0 mmol L^−1^ or new use of hypoglycaemic drug therapy	Mean 6.8 years 7 cases	Criteria 1 Model 1: RR = 2.40 (0.94, 6.12) Model 2: RR = 2.19 (0.85, 5.60) Reference: BMI < 25 kg m^−2^ without metabolic syndrome Criteria 2 Model 1: RR = 3.79 (1.66, 8.62) Model 2: RR = 3.28 (1.44, 7.50) Reference: BMI < 25 kg m^−2^ and insulin sensitive	Model 1: Age, sex Model 2: Further adjusted for family history of diabetes, IGT	5
Hadaegh *et al*. 2011 (30)	*n* = 5,250 ≥20 years 41.6% male Free of diabetes Tehran Nationally representative	452 (37.5%)	≤2 of: waist circumference ≥ 94.5 cm, HDL-cholesterol < 1.04 mmol L^−1^ in men and <1.03 mmol L^−1^ in women, triglycerides ≥ 1.7 mmol L^−1^ or lipid-lowering drug use, blood pressure ≥ 130/85 mmHg or hypertension treatment, fasting glucose ≥ 5.5 mmol L^−1^ or previously diagnosed diabetes Obese BMI ≥ 30 kg m^−2^	Fasting plasma glucose ≥ 7 mmol L^−1^ or 2 h post-challenge plasma glucose ≥ 11.1 mmol L^−1^ or diabetes medication use	6.5 years 7 cases (men)† 11 cases (women)†	Men Model 1: OR = 3.80 (1.70, 8.90) Model 2: OR = 3.60 (1.50, 8.40) Women Model 1: OR = 2.20 (1.00, 4.70) Model 2: OR = 2.20 (1.00, 4.70) Reference: BMI < 25 kg m^−2^, metabolically healthy	Model 1: Age Model 2: Further adjusted for family history of diabetes, history of CVD, education, smoking, lifestyle intervention received	7
Arnlov *et al*. 2011 (33)	*n* = 1,675 50 years 100% male Free of type 2 diabetes Sweden Community-based	28 (31.8%)	Modified NCEP ATP3 ≤2 of: fasting blood glucose ≥ 5.6 mmol L^−1^ (corresponding to fasting plasma glucose ≥ 6.1 mmol L^−1^), blood pressure ≥ 130/85 mmHg or treatment, triglycerides ≥ 1.7 mmol L^−1^, HDL-cholesterol < 1.04 mmol L^−1^, BMI ≥ 29.4 kg m^−2^ BMI > 30 kg m^−2^	Fasting blood glucose ≥ 6.1 mmol L^−1^ (corresponding to fasting plasma glucose ≥ 7.0 mmol L^−1^) or use of anti-diabetes medication	20 years 9 cases	Whole sample (*n* = 1,675) Crude OR = 12.15 (5.10, 28.96) Adjusted OR = 11.73 (4.88, 28.16) Sensitivity: Normal fasting glucose at baseline (*n* = 1,541) Crude OR = 13.35 (5.55, 32.11) Adjusted OR = 13.19 (5.42, 32.09) Reference: BMI < 25 kg m^−2^ without metabolic syndrome	Age, smoking, physical activity	6
Kim *et al*. 2012 (35)	*n* = 8,748 20–79 years 65.2% male Free of self-reported history of physician diagnosed diabetes, or taking anti-hyperglycaemic medication, fasting blood glucose ≥ 126 mg dL^−1^, HbA1c ≥6.5% Korea Clinic-based	59 (41%)	≤2 of: fasting plasma glucose ≥ 100 mg dL^−1^ or anti-diabetic treatment, blood pressure ≥ 130/85 mmHg, or anti-hypertensive treatment, plasma triglycerides ≥ 150 mg dL^−1^, plasma HDL-cholesterol < 40 mg dL^−1^ in men and <50 mg dL^−1^ in women, waist circumference ≥ 90 cm in men and ≥80 cm in women. BMI ≥ 30 kg m^−2^ and repeated with Asian specific cut-off – BMI ≥ 27.5 kg m^−2^	Fasting plasma glucose ≥ 126 mg dL^−1^, HbA1c > 6.5%, or on anti-hyperglycaemic medication	5 years 5 cases	Crude OR = 5.31 (2.08, 13.56) Adjusted OR = 4.93 (1.90, 12.79) Reference: BMI < 25 kg m^−2^ metabolically healthy Asian-specific Crude OR = 4.57 (2.57, 8.10) Adjusted OR = 4.31 (2.36, 7.86) Reference: BMI < 23 kg m^−2^ metabolically healthy	Age, sex, smoking, alcohol, physical activity	7
Hwang *et al*. 2012 (34)	*n* = 1,547 18–59 years 40.7% male Free of type 2 diabetes, hypertension, history of stroke and metabolic abnormalities except central adiposity Taiwan Nationally representative	38 (28.5%)	AHA criteria modified for Asian populations ≤2 of: waist circumference ≥ 90 cm for men and ≥80 cm for women, triglycerides ≥ 1.7 mmol L^−1^, HDL-cholesterol < 1.0 mmol L^−1^ for men and <1.3 mmol L^−1^ for women, systolic blood pressure ≥ 130 mmHg or diastolic blood pressure ≥ 85 mmHg or current use of anti-hypertensive drugs, and fasting plasma glucose ≥ 5.6 mmol L^−1^ or current use of anti-hyperglycaemic drugs BMI ≥ 27 kg m^−2^	Fasting plasma glucose ≥ 7.0 mmol L^−1^ or HbA1C > 6.5% or use of anti-hyperglycaemic mediation	Mean 5.4 years 3 cases (men)† 5 cases (women)†	Men HR = 14.30 (1.21, 168.00) Women HR = 14.60 (3.23, 65.50) Total (men and women) HR = 11.50 (3.38, 39.10) Reference: BMI 18.5–22.9 kg m^−2^ metabolically healthy	Age, smoking status, alcohol intake, exercise, family history of diabetes or hypertension	8
Soriguer *et al*. 2013 (37)	*n* = 1,051 18–65 years 37.7 % male Free of type 2 diabetes Spain Nationally representative	105 (48.4%)	HOMA-IR < 90th percentile, triglycerides < 150 mg dL^−1^, fasting glucose < 110 mg dL^−1^ BMI ≥ 30 kg m^−2^	Fasting plasma glucose ≥ 7 mmol L^−1^	6 and 11 years 17 cases (6-year follow-up) 11 cases (11-year follow-up)	After 6 years Model 1: OR = 3.62 (1.83, 7.17) Model 2: OR = 2.16 (1.07, 4.36) After 11 years Model 1: OR = 6.76 (2.58, 17.69) Model 2: OR = 4.12 (1.82, 9.34) Reference: metabolically healthy BMI < 30 kg m^−2^	Model 1: Unadjusted Model 2: Age, sex, weight change, abnormal glucose regulation (IFG, IGT)	4
Appleton *et al*. 2013 (29,30)	*n* = 3,743 ≥18 years 39% male (MHO group) Free of CVD/stroke and not underweight Australia Nationally representative	454 (44.2%)	<2 IDF metabolic syndrome criteria: triglycerides ≥ 1.7 mmol L^−1^, HDL-cholesterol < 1 mmol L^−1^ in men or <1.3 mmol L^−1^ in women or lipid-lowering medication use, blood pressure ≥ 130/85 mmHg or anti-hypertensive medication use, fasting glucose ≥ 5.6 mmol L^−1^ or self-reported diabetes BMI ≥ 30 kg m^−2^	Self-reported doctor diagnosis or fasting plasma glucose ≥ 7 mmol L^−1^	Median 8.2 years 11 cases	OR = 2.09 (0.87, 5.03) Reference: metabolically healthy BMI 18.5–24.9 kg m^−2^)	Age, sex, household income, family history of diabetes	6
ELSA 2013	*n* = 3,066 Mean age 64.6 43.3% male Free from physician diagnosed diabetes England Nationally representative	308 (38.3%)	<2 of: hypertension risk (clinic BP > 130/85 mmHg, or hypertension diagnosis, or use of anti-hypertensive medication); diabetes risk (HbA1c > 6%); low-grade inflammation (CRP ≥ 3 mg L^−1^); adverse HDL-cholesterol profile (<1.03 mmol L^−1^ in men and <1.30 mmol L^−1^ women); adverse triglycerides (≥1.7 mmol L^−1^). BMI ≥ 30 kg m^−2^	Self-reported physician diagnosis, based upon fasting plasma glucose ≥ 7 mmol L^−1^	Mean 5.9 years 12 cases	Model 1: HR = 9.30 (2.60, 32.70) Model 2: HR = 8.60 (2.40, 30.40) Reference: metabolically healthy BMI < 25 kg m^−2^)	Model 1: Age, sex Model 2: Further adjusted for cigarette smoking, frequency of alcohol intake, physical activity, wealth, depressive symptoms	7

* Study quality assessed according to the rigor of study exposure, outcome and model adjustment strategy. Points were assigned as follows: 2 points if the study considered metabolic clustering; 1 point if the study considered insulin profile alone; 2 points if diabetes diagnosis was based upon objective clinical measurement (i.e. blood glucose level); 1 point if diabetes diagnosis was based upon self-report only; 1 point if each of the following covariates were considered: family history of diabetes, ethnicity, alcohol consumption, smoking status, physical activity, dietary sugar intake and socioeconomic status. Studies were scored out of 11 possible points.

† Estimated from published cumulative incidence (%) figure.

AHA, American Heart Association; BMI, body mass index; CI, confidence interval; CRP, C-reactive protein; CVD, cardiovascular disease; HDL, high-density lipoprotein; HOMA-IR, homeostasis model assessment-estimated insulin resistance; HR, hazard ratio; IDF, International Diabetes Federation; IFG, impaired fasting glucose; IGT, impaired glucose tolerance; MHO, metabolically healthy but obese; NCEP, National Cholesterol Education Program; OR, odds ratio; RR, relative risk.

**Figure 2:**
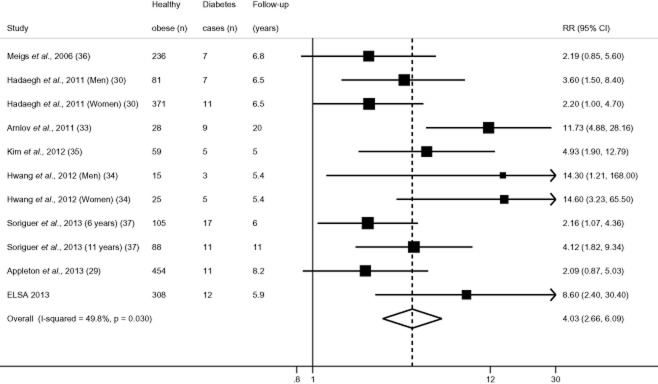
Metabolically healthy obesity and adjusted relative risk (RR) of incident type 2 diabetes.

**Figure 3:**
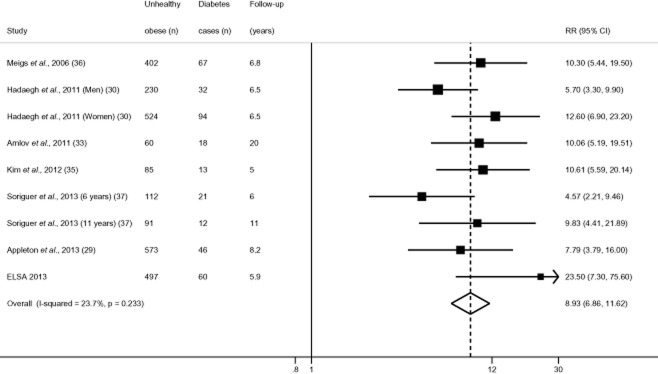
Metabolically unhealthy obesity and adjusted relative risk (RR) of incident type 2 diabetes. Note: Analysis excludes Hwang *et al*. (2012) (34) as authors considered metabolically healthy participants at baseline only.

The reference category was a metabolically healthy non-obese group in all studies; however, specific cut-offs varied, with one study using a broad ‘non-obese’ group as the reference (BMI < 30 kg m^−2^) (37), whereas ELSA and others excluded overweight individuals from the reference group by setting the cut-off as BMI < 25 kg m^−2^ (30,33,35,36). Still others excluded both overweight and underweight adults in their reference group (29,34). Overall, there appeared to be wide variability in effect estimates for type 2 diabetes, ranging from 2.09 (95% CI = 0.87–5.05) in Appleton *et al*. (29) to 14.60 (95% CI = 3.23–65.50) in Hwang *et al*. (women) (34). However, all RR estimates exceeded one, with none reporting a reduced risk of type 2 diabetes in metabolically healthy obese adults. Figure 2 presents results of the random effects meta-analysis, which modelled the log of ORs, risk ratios, HR and CIs pooled from respective studies. The summary RR was 4.03 (95% CI = 2.66–6.09), suggesting that the healthy obese had over four times higher risk of incident type 2 diabetes than the healthy normal-weight group. In comparison, the corresponding pooled RR in the metabolically unhealthy obese group was 8.93 (95% CI = 6.86–11.62) (Fig. 3). There was variability in the effect size (*I*^2^ statistic of 49.8%; *P* = 0.03), although RRs for healthy obesity exceeded one in every study. We performed meta-regression to test the extent to which specific study-level factors explained the between-study heterogeneity in effects, chosen *a priori* (39) as age, ethnicity, length of follow-up and study quality. However, no study reported the ethnic composition of their analytical sample, preventing us from exploring contributions of that factor. Neither study quality (*P* = 0.18), length of follow-up (*P* = 0.34) nor age (*P* = 0.99) significantly predicted heterogeneity in effect estimates.

## Discussion

In the identified studies, metabolically healthy obese adults showed, with few exceptions, a substantially increased risk for developing type 2 diabetes compared with metabolically healthy normal-weight adults. When prospective evidence was synthesized in a random effects meta-analysis (average length of follow-up ranging from 5 years in Kim *et al*. (35) to 20 years in Arnlov *et al*. (33)), metabolically healthy obese adults demonstrated over four times greater risk of developing type 2 diabetes over time when compared to healthy normal weight adults; albeit the risk among the healthy obese was approximately half that of the unhealthy obese group.

Our original analysis of older English adults suggests that after adjustment for covariates, the healthy obese were still at significantly increased risk for incident type 2 diabetes, albeit to a lesser extent than their unhealthy obese counterparts. These findings are consistent with other studies accounting for similar baseline covariates (30,34), and the commonly observed trend of increasing risk across BMI groups further supports body mass as an important risk factor. Healthy obese adults did not show a statistically significant increased risk of type 2 diabetes compared with healthy normal-weight adults in an early American study (36), and in a more recent Australian study (29), although in both of these studies, the RR exceeded one.

We observed heterogeneity in effects of healthy obesity between studies, with estimates ranging from 2.09 (95% CI = 0.87–5.05) in Appleton *et al*. (29) to 14.60 (95% CI = 3.23–65.50) in Hwang *et al*. (women) (34). There was no evidence to suggest that this was explained by differences in participant's age, duration of follow-up or study quality. Such heterogeneity might stem from variations in phenotype criteria employed across studies, such as inconsistencies in metabolic factors considered and specific cut-points used. However, despite this heterogeneity, all estimates appeared to be in the same direction, with no studies reporting a reduced risk of incident type 2 diabetes for healthy obese adults.

Large CIs were observed across several studies, pointing to the relatively large degree of uncertainly attached to respective estimates. While sample numbers of metabolically healthy obese individuals are often small in population-based prospective studies (ranging from *n* = 15 in Hwang *et al*. [men] (34) to several hundred in others), this phenotype does not represent a small segment of the population. Nearly one-third of obese adults in the general population are thought to be metabolically healthy (14,15), which, as the proportion of obese adults increases, will continue to represent a substantial number of people in both relative and absolute terms. Small sample sizes in previous studies may reflect the research challenges associated with studying repeat clinical characteristics over long periods of time.

Type 2 diabetes is often regarded as a state of chronic fuel surfeit (40), and as such, dietary factors are expected to play a central role in disease risk. Despite this, no studies considered the influence of dietary factors, such as sugar intake, on the risk of type 2 diabetes for healthy obese adults. Likewise, only half of the studies considered any indicator of cardiorespiratory fitness or physical activity, which are also important protective factors against type 2 diabetes development (10,11). Additionally, a limited range of prescription drugs were considered in statistical adjustments. Whereas the healthy obese phenotype is often defined according to use of anti-hyperglycaemic or anti-hypertensive medications, the use of statins was considered in only two studies (29,30). Other prescription drug use or dietary interventions may help account for their apparent metabolic protection and should be considered in more depth in future work.

Potential confounding effects of environmental factors are also notably absent from the evidence base. For example, living in a deprived residential environment is associated with an increased risk of obesity and type 2 diabetes (41–43), which may be explained in part by increased accessibility of energy-dense/nutrient-poor foods, decreased opportunities for recreational or transport-based physical activity (44), and psychosocial stress (45,46). Environmentally distributed chemicals, known as persistent organic pollutants, have also been positively associated with the risk of type 2 diabetes among obese and non-obese adults in a dose-response manner (47), and thus could potentially confound associations between obesity and incident type 2 diabetes. Until such factors are adequately controlled for, it remains difficult to separate their effects from direct effects of healthy obesity, and thus make firm conclusions regarding chronic disease risk.

A critical factor to consider when estimating future disease risk in the healthy obese group is the stability of metabolic health over time – i.e. whether metabolically healthy obese adults actually remain metabolically healthy for the duration of follow-up, or whether they transition into an unhealthy state before outcomes are assessed. Evidence on long-term phenotypic stability as it relates to diabetes risk is currently limited. However, one recent study found that obese adults who maintained metabolic health for up to 10 years showed no increased risk for type 2 diabetes incidence (29). Sustained metabolic health was associated with younger age and lower levels of abdominal adiposity as indicted by lower waist circumferences (29). The accuracy of diabetes risk estimates would be improved by paying closer attention to stability and change in metabolic health over time in both obese and non-obese populations.

It is also important to note that despite an increased risk for incident type 2 diabetes compared with healthy normal-weight adults, healthy obese adults often show a lower risk for type 2 diabetes than metabolically unhealthy groups of any body mass. For example, in our analysis of ELSA, the healthy obese demonstrated an 8.6 (95% CI = 2.4–30.4) times higher risk of developing diabetes, whereas the unhealthy normal-weight showed a higher risk for disease development at 9.9 (95% CI = 2.9–36.7) after adjusting for baseline socioeconomic, health and behavioural covariates. The increased risk in metabolically unhealthy normal-weight adults is apparent when the phenotype is defined by either metabolic clustering or insulin resistance only (36). Greater attention should be paid to unhealthy normal-weight adults as they represent a sizable proportion of the general population and may be less targeted for interventions.

Intriguingly, standard weight-loss interventions among the healthy obese have experienced limited success. For example, healthy obese adults showed no improvement in individual metabolic risk factors such as blood lipids, inflammatory markers (48) and insulin sensitivity (49) in response to diet- and/or exercise-based interventions, whereas others reported detrimental effects such as decreased insulin sensitivity (50). Another study reports that healthy obese adults who lost fat mass up to the point of resistance to further weight-loss experienced notable adverse physiological effects including worsened appetite regulation, decreased energy expenditure and increased depressive symptoms (51), all of which may promote weight regain. It remains unknown whether such adverse physiological effects are characteristic of the entire healthy obese population or only a subgroup, as no studies to date have utilized nationally representative data. ‘Weight loss’ is also a crude metric in light of emerging evidence showing more favourable fat distribution in the healthy obese, characterized by lower visceral fat and greater thigh subcutaneous fat (52), along with favourable adipose tissue function and morphology (53). Targeted fat loss may therefore be more appropriate for healthy obese adults. Indeed, several studies report reductions in visceral fat among healthy obese men and women (49,54,55), whereas others show increased levels of cardiorespiratory fitness (54), improved insulin sensitivity and improved fasting insulin (55), all of which may contribute to a lower risk of type 2 diabetes.

### Strengths and limitations

This is the first meta-analysis to summarize the risk of incident type 2 diabetes in the metabolically healthy obese phenotype. These data will help establish whether an apparently healthy subset of the obese population faces an increased risk for metabolic disease. We had the advantage of supplementing this meta-analysis with an original estimate from a nationally representative sample of older adults in England, affording a larger sample size and a more complete view of diabetes risk across adulthood. This study also explored the potential impact of relevant confounding factors, such as age, duration of follow-up and study quality using meta-regression.

The between-study heterogeneity in effects observed may reflect differences in study characteristics such as phenotype definitions, length of follow-up, statistical adjustment strategies, as well as differences inherent to populations such as age, ethnicity or obesity management strategies such as lifestyle interventions or prescription drug use. However, with the small number of studies currently available, and with each measuring a different population, numbers within each age or ethnic group would likely be too small to draw meaningful conclusions about the source of heterogeneity. A standard definition of what constitutes ‘metabolic health’ within obese populations would aid efforts to understand differences in effects due purely to specific demographic or lifestyle factors.

## Conclusion

Metabolically healthy obese adults show a substantially increased risk of incident type 2 diabetes compared with metabolically healthy normal-weight adults. Existing prospective evidence does not indicate that healthy obesity is a harmless condition.

## Supporting information

Appendix S1 Characteristics of the ELSA study population at baseline (*n* = 3,066)

Appendix S2 Metabolically healthy obesity and risk of incident type 2 diabetes over 6 years follow-up in ELSA. Participants free from physician diagnosed diabetes at baseline (*n* = 3,066)

Appendix S3 Metabolically healthy obesity and risk of incident diabetes over 6 years follow-up in ELSA. Participants free from physician diagnosed diabetes at baseline
