# Supplementary material for: Metabolically healthy obesity and risk of incident type 2 diabetes: a meta-analysis of prospective cohort studies
Source: Obes Rev. 2014 Mar 24;15(6):504–15. doi: 10.1111/obr.12157 (PMC4309497; doi:10.1111/obr.12157)
Supplement: Appendix S1 — Characteristics of the ELSA study population at baseline (n = 3,066) [file obr0015-0504-sd1.docx]

**Appendix 1:** Characteristics of the ELSA study population at baseline (N=3,066)

|  | **Metabolically healthy**  **non-obese (n=1561)** | **Metabolically unhealthy non-obese**  **(n=700)** | **Metabolically healthy**  **obese**  **(n=308)** | **Metabolically unhealthy obese**  **(497)** |
| --- | --- | --- | --- | --- |
| Age (yrs) | 64.2±8.4 | 66.4±8.9^a^ | 62.9±7.8 | 64.5±8.3 |
| Men (%) | 43.2 | 47.9 | 40.9^c^ | 38.4^c^ |
| Depressive symptoms (% CES-D>3) | 9.9 | 11.0 | 12.7 | 15.3^a^ |
| Current smokers (%) | 10.7 | 17.7 | 7.5^a^ | 13.7 |
| Alcohol (% daily consumption) | 29.0 | 22.9 | 23.1 | 16.3^a^ |
| Vigorous physical activity (% at least once/wk) | 40.2^a^ | 29.9 | 33.8 | 24.3^a^ |
| HDL cholesterol (mmol/l) | 1.68±0.37^a^ | 1.43±0.36 | 1.55±0.30^a^ | 1.38±0.32 |
| Triglycerides (mmol/l) | 1.34±0.70^b^ | 2.28±1.08 | 1.50±0.75^b^ | 2.38±1.35 |
| Body mass index (kg/m^2^) | 25.1±2.6^a^ | 26.4±2.3^a^ | 32.7±2.9^a^ | 34.1±4.0^a^ |
| HbA1c (%) | 5.36±0.33^a^ | 5.55±0.57^a^ | 5.45±0.42^a^ | 5.67±0.68^a^ |
| Systolic BP (mmHg) | 129.6±17.0^b^ | 138.8±18.7 | 134.4±15.3^b^ | 140.4±18.3 |
| Diastolic BP (mmHg) | 73.5±9.6^a^ | 77.1±11.9 | 76.8±8.0 | 80.0±11.4^a^ |
| C-reactive protein (mg/l)* | 2.34±1.47^a^ | 4.07±1.96^a^ | 3.13±1.55^a^ | 4.62±2.04^a^ |

Values are means ± SD unless otherwise stated. *Geometric mean.

^a^ significantly different (p<0.05) compared with all other groups; ^b^ significantly different compared with metabolically unhealthy groups; ^c^ significantly different compared with non-obese groups.

**Obesity** defined as BMI ≥ 30 kg/m^2^; **Metabolically unhealthy** defined as ≥ 2 metabolic risk factors, including, hypertension risk (clinic BP >130/85 mmHg, or hypertension diagnosis, or use of anti-hypertensive medication), diabetes risk (HbA1c > 6%), low grade inflammation (CRP≥ 3mg/l), adverse HDL cholesterol profile (<1.03 mmol/l in men and <1.30 mmol/l women), adverse triglycerides (≥ 1.7 mmol/l).
