# Supplementary material for: Metabolically healthy obesity and risk of incident type 2 diabetes: a meta-analysis of prospective cohort studies
Source: Obes Rev. 2014 Mar 24;15(6):504–15. doi: 10.1111/obr.12157 (PMC4309497; doi:10.1111/obr.12157)
Supplement: Appendix S2 — Metabolically healthy obesity and risk of incident type 2 diabetes over 6 years follow-up in ELSA. Participants free from physician diagnosed diabetes at baseline (n = 3,066) [file obr0015-0504-sd2.docx]

**Appendix 2:** Metabolically healthy obesity and risk of incident type 2 diabetes over 6 years follow-up in ELSA. Participants free from physician diagnosed diabetes at baseline (N=3,066)

|  | Cases/N | **Model 1**  Hazard Ratio  (95% CI) | **Model 2**  Hazard Ratio  (95% CI) |
| --- | --- | --- | --- |
| Metabolically healthy/ BMI < 25 | 3/702 | Reference | Reference |
| Metabolically unhealthy/ BMI<25 | 9/177 | 11.3 (3.0, 41.9) | 9.9 (2.9, 36.7) |
| Metabolically healthy/ BMI 25<30 | 11/856 | 2.9 (0.8, 10.2) | 2.9 (0.8, 10.3) |
| Metabolically unhealthy/ BMI 25<30 | 43/520 | 18.4 (5.7, 59.3) | 16.7 (5.2, 54.2) |
| Metabolically healthy/ BMI 30+ | 12/308 | 9.3 (2.6, 32.7) | 8.6 (2.4, 30.4) |
| Metabolically unhealthy/ BMI 30+ | 60/497 | 29.5 (9.2, 94.0) | 23.5 (7.3, 75.6) |
| *p-trend* |  | <0.001 | <0.001 |

**Model 1** adjusted for age and sex.

**Model 2** adjusted for age, sex, cigarette smoking (current, previous or non-smoker), frequency of alcohol intake (5 or more times per wk, once per wk, once every couple of months, rarely or never), physical activity (none, moderate activity at least once a week, vigorous activity at least once a week), wealth quintile, depressive symptoms (CES-D> 3).
