# Supplementary material for: Metabolically healthy obesity and risk of incident type 2 diabetes: a meta-analysis of prospective cohort studies
Source: Obes Rev. 2014 Mar 24;15(6):504–15. doi: 10.1111/obr.12157 (PMC4309497; doi:10.1111/obr.12157)
Supplement: Appendix S3 — Metabolically healthy obesity and risk of incident diabetes over 6 years follow-up in ELSA. Participants free from physician diagnosed diabetes at baseline [file obr0015-0504-sd3.docx]

**Appendix 3:** Metabolically healthy obesity and risk of incident diabetes over 6 years follow-up in ELSA. Participants free from physician diagnosed diabetes at baseline

|  | Cases/N | **Model 1**  Hazard Ratio  (95% CI) | **Model 2**  Hazard Ratio  (95% CI) |
| --- | --- | --- | --- |
| Metabolically healthy/ BMI < 25 | 1/404 | Reference | Reference |
| Metabolically unhealthy/ BMI<25 | 11/475 | 8.7 (1.1, 87.3) | 7.1 (0.9, 55.6) |
| Metabolically healthy/ BMI 25<30 | 1/344 | 1.1 (0.1, 17.8) | 1.0 (0.1, 17.1) |
| Metabolically unhealthy/ BMI 25<30 | 53/1032 | 19.1 (2.6, 138.2) | 16.4 (2.3, 119.2) |
| Metabolically healthy/ BMI 30+ | 2/83 | 9.8 (0.9, 108.5) | 8.5 (0.8, 93.6) |
| Metabolically unhealthy/ BMI 30+ | 70/721 | 39.4 (5.5, 284.0) | 29.3 (4.1, 212.1) |
| *p-trend* |  | <0.001 | <0.001 |

*Obesity defined as BMI ≥ 30 kg/m2;* ***Metabolically unhealthy defined as ≥ 1 metabolic risk factors****, including, hypertension risk (clinic BP >130/85 mmHg, or hypertension diagnosis, or use of anti-hypertensive medication), diabetes risk (HbA1c > 6%), low grade inflammation (CRP≥ 3mg/l), adverse HDL cholesterol profile (<1.03 mmol/l in men and <1.30 mmol/l women), adverse triglycerides (≥ 1.7 mmol/l).*

**Model 1** adjusted for age and sex.

**Model 2** adjusted for age, sex, cigarette smoking (current, previous or non-smoker), frequency of alcohol intake (5 or more times per wk, once per wk, once every couple of months, rarely or never), physical activity (none, moderate activity at least once a week, vigorous activity at least once a week), wealth quintile, depressive symptoms (CES-D> 3).
